# Extrauterine adenomyoma of the liver: A case report with a review of the literature

**DOI:** 10.1016/j.radcr.2026.09.036

**Published:** 2026-10-03

**Authors:** Behnam Parandoush, Hadi Koohi, Niloofar Ayoobi Yazdi, Amir Kasraianfard, Masoomeh Safaei, Faeze Salahshour

**Affiliations:** a Department of Radiology, Advanced Diagnostic and Interventional Radiology Research Center, Imam Khomeini Hospital Complex, Tehran University of Medical Sciences, Tehran, Iran; b Hepatobiliary Surgery and Liver Transplantation Division, Department of General Surgery, Imam Khomeini Hospital Complex, Tehran, Iran; c Department of Pathology, Cancer Institute, Imam Khomeini Hospital Complex٫ Tehran University of Medical Sciences, Tehran, Iran

**Keywords:** Extrauterine adenomyoma, Liver, Adenomyosis, Endometriosis, Diagnosis, Differential, Magnetic resonance imaging

## Abstract

Extrauterine adenomyoma is an exceedingly rare benign lesion composed of endometrial glands/stroma embedded within smooth muscle bundles and located outside the uterus. The majority of extrauterine adenomyomas occur in the pelvis involving the ovary, rectovaginal space, or broad ligament; hepatic involvement is extremely rare. We present the case of a 47-year-old woman with a subcapsular mass in segment VI of the liver that was detected during the work-up for abdominal pain. Initial core needle biopsy of the mass was suggestive of FNH or hepatic adenoma, with expert consultation favoring parasitic leiomyoma. Finally, the patient underwent surgical resection, and the histopathological exam revealed endometrial glands and stroma, admixed with smooth muscle, confirming the diagnosis of extrauterine adenomyoma. This case highlights the diagnostic challenge of extrauterine adenomyoma and emphasizes the importance of considering this entity in the differential diagnosis of pelvic or extrapelvic masses, particularly in women with a history of gynecologic lesions. Recognizing this rare condition may help prevent misdiagnosis and inappropriate management.

## Introduction

Adenomyoma is a benign lesion characterized by the presence of endometrial glands and stroma embedded within hypertrophic smooth muscle [1]. Although adenomyomas are typically confined to the uterus, rare cases of extrauterine adenomyoma have been reported. These lesions most commonly occur in the pelvis, including the ovary, broad ligament, and rectovaginal septum, but have also been described at unusual extrapelvic sites, including the liver.

The exact pathogenesis remains uncertain. Proposed mechanisms include implantation of endometrial tissue followed by smooth muscle proliferation, Müllerian duct fusion defects, and metaplastic transformation of subcoelomic mesenchyme [2,3].

Because of their rarity and nonspecific clinical and imaging findings, extrauterine adenomyomas may mimic leiomyomas, endometriotic implants, or even malignant neoplasms, making preoperative diagnosis challenging. Cross-sectional imaging findings are usually nonspecific; therefore, definitive diagnosis generally relies on histopathological examination [4]. Given the potential for diagnostic confusion, recognition of this entity is important to avoid unnecessary intervention or inappropriate management. We report a rare case of hepatic extrauterine adenomyoma with a focused review of all previously reported hepatic cases, emphasizing the imaging findings, radiologic differential diagnosis, and radiologic-pathologic correlation.

## Case presentation

A 47-year-old woman with a history of uterine fibroma surgery 5 years earlier was referred to our clinic for evaluation of persistent abdominal pain. Her previous surgical history included an umbilical hernia repair 1 year earlier, a cholecystectomy 15 years ago, and two cesarean sections. Routine laboratory examinations were unremarkable. Abdominal and pelvic ultrasonography performed for evaluation of abdominal pain revealed mild hepatic steatosis and a hypoechoic mass measuring 57×45 mm in the posterior aspect of the right hepatic lobe. Pelvic ultrasonography demonstrated an enlarged myomatous uterus measuring 150×93 mm, containing multiple large subserosal and pedunculated myomas, with the largest measuring up to 97×70 mm. Contrast-enhanced computed tomography (CT) of the abdomen and pelvis (Fig. 1) revealed an enlarged myomatous uterus with large subserosal and pedunculated fibroids, some showing cystic degeneration. In addition, a 51 mm heterogeneously enhancing lesion was identified in segment VI of the liver. The lesion was subcapsular in location, formed an obtuse angle with the liver capsule, and demonstrated a small exophytic component extending into Morrison’s pouch (Fig. 2).

**Fig. 1:**
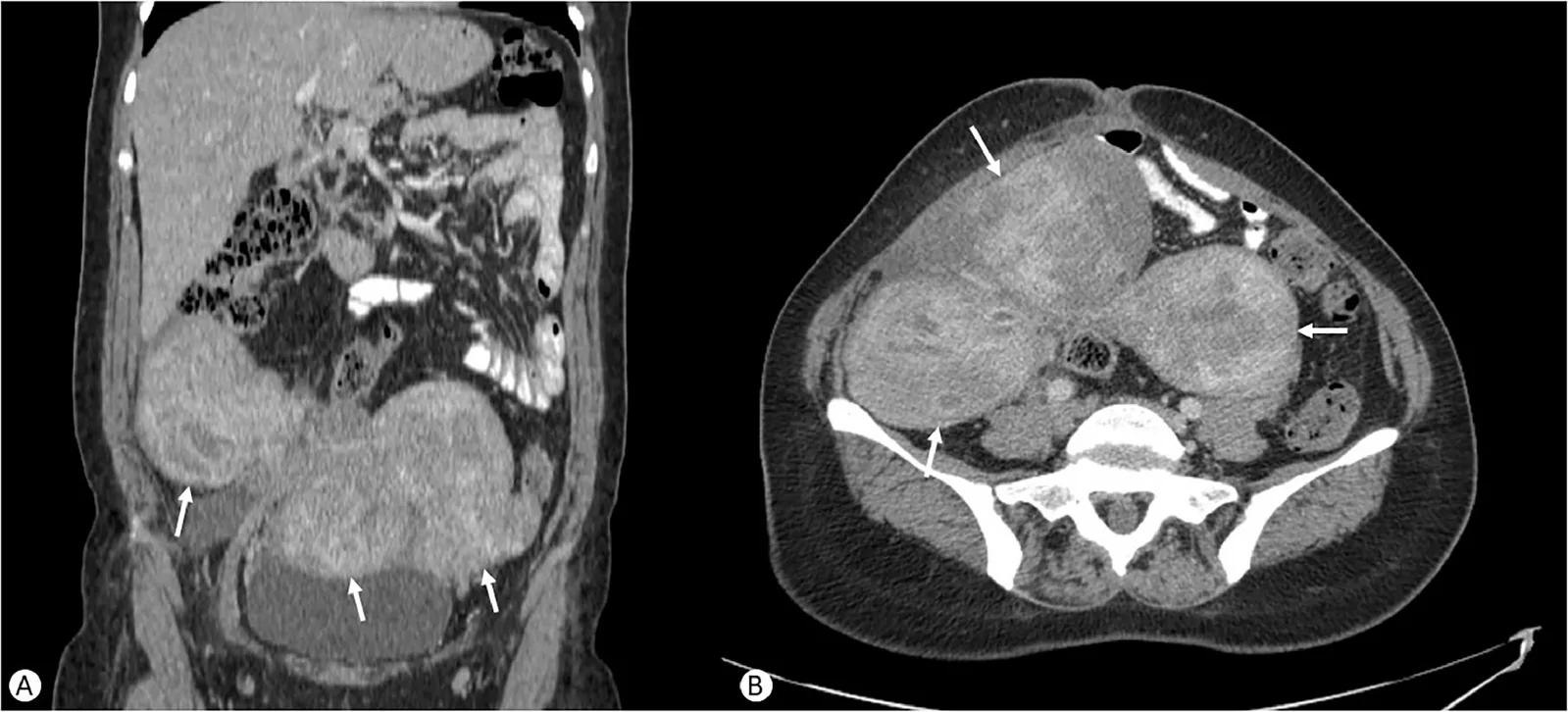
Contrast-enhanced CT scan of the abdomen and pelvis in coronal (A) and axial (B) planes shows multiple pedunculated myomas (arrows), containing multiple small cystic foci.

**Fig. 2:**
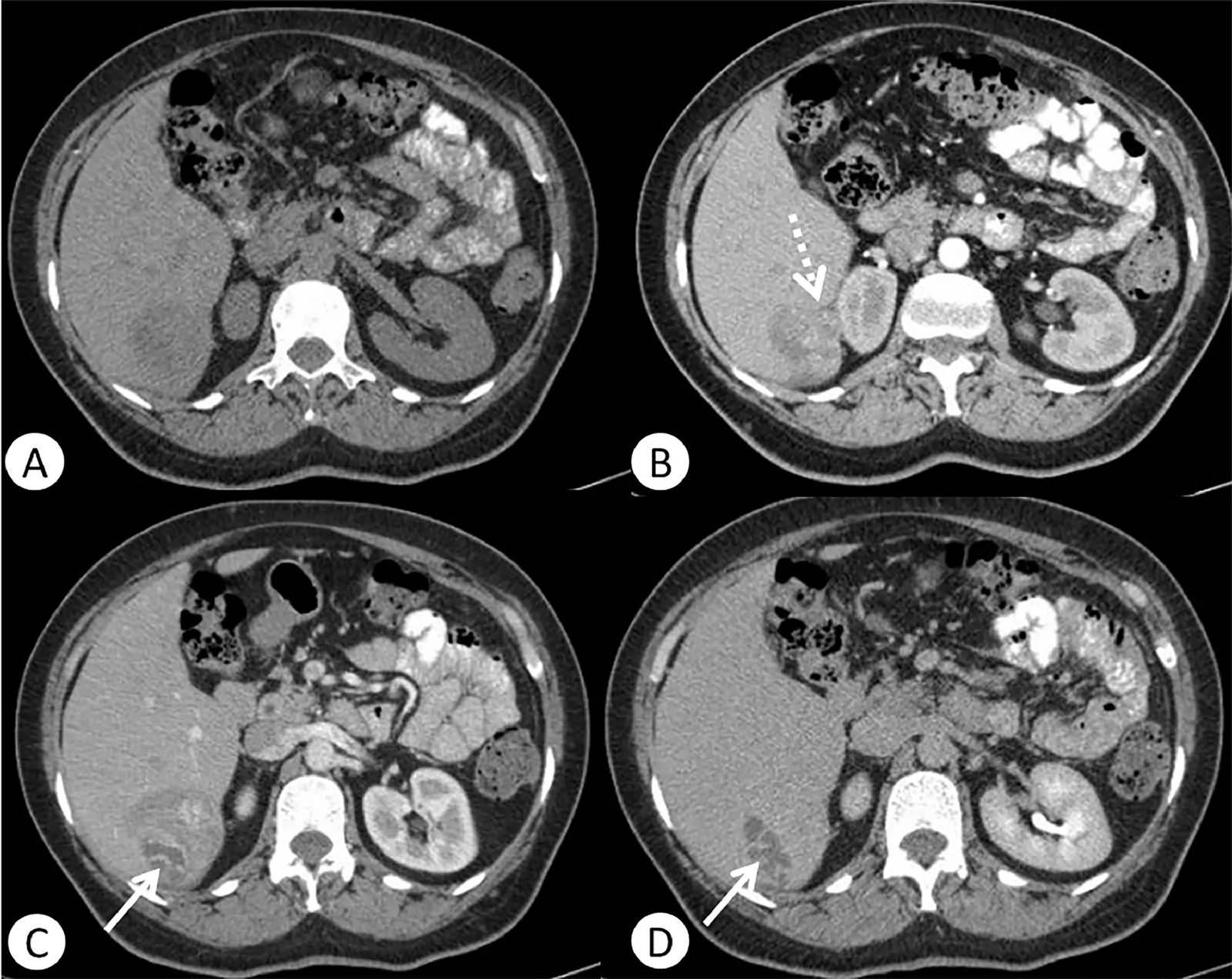
Axial non-contrast (A), arterial (B), portal (C), and delayed (D) phases of contrast-enhanced abdominal CT scan reveal a round, heterogeneously enhancing lesion in the subcapsular aspect of segment VI of the liver, containing multiple small cystic components (solid arrows). The mass forms an obtuse angle with the liver capsule (dotted arrow) and demonstrates persistent enhancement through the portal venous and delayed phases.

Pelvic Magnetic Resonance Imaging (MRI) confirmed the enlarged uterus with multiple exophytic myomas, occupying the pelvis and lower abdomen (Fig. 3). Dynamic liver MRI demonstrated a well-defined subcapsular lesion in segment VI that was hypointense on T1-weighted images and hyperintense on T2-weighted images, with persistent enhancement from the arterial through delayed phases. T1 in-phase GRE images also revealed susceptibility/signal dropout compatible with hemosiderin deposition (Fig. 4). Follow-up dynamic MRI performed 13 months later demonstrated interval enlargement of the lesion with development of multiple hemorrhagic and cystic components (Fig. 5).

**Fig. 3:**
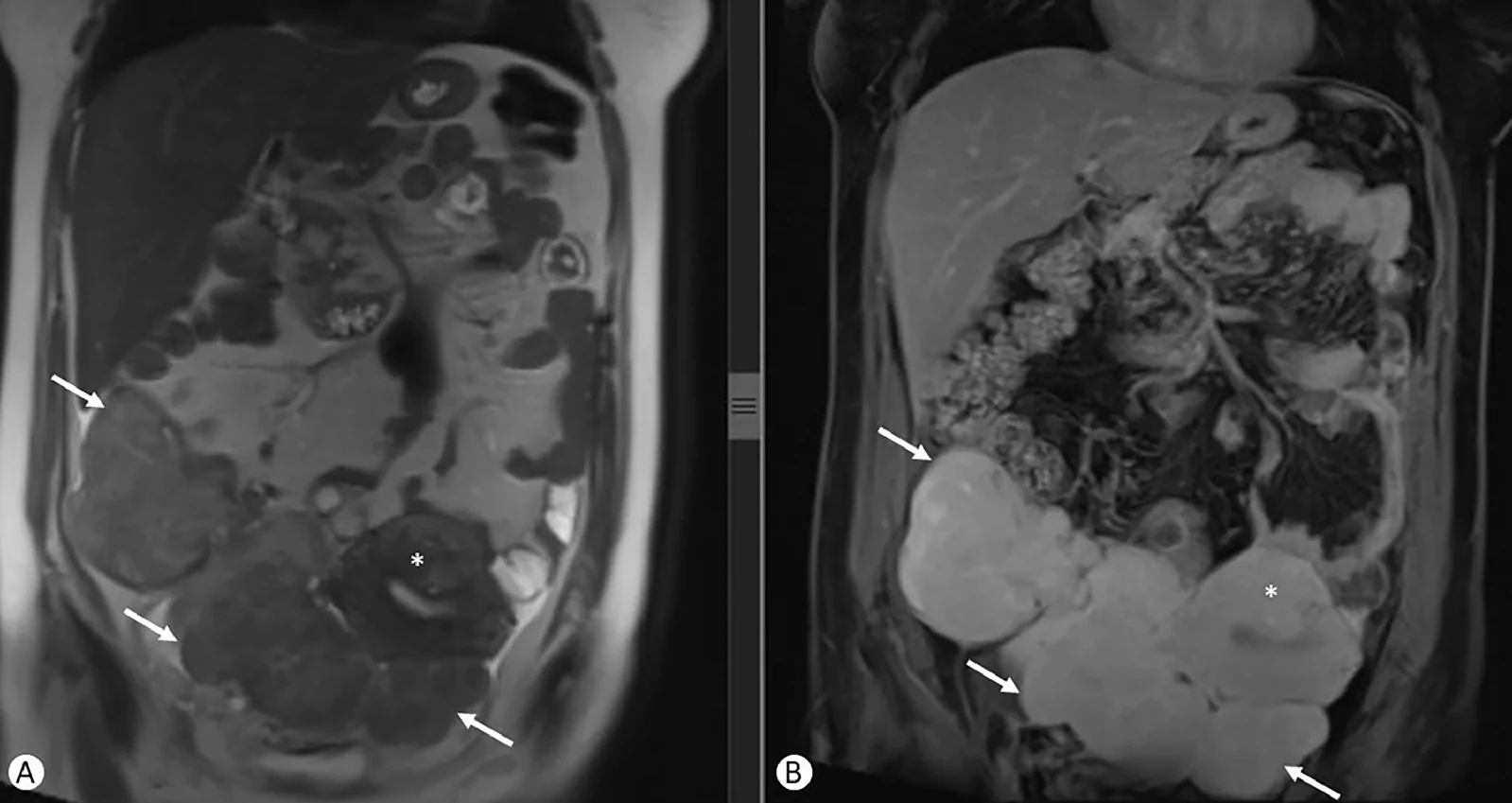
Coronal T2 (A) and post-gadolinium fat-saturated T1 (B) MRI of the abdomen and pelvis show the enlarged uterus (*) and multiple enhancing pedunculated myomas (arrows) in the pelvis and lower abdomen.

**Fig. 4:**
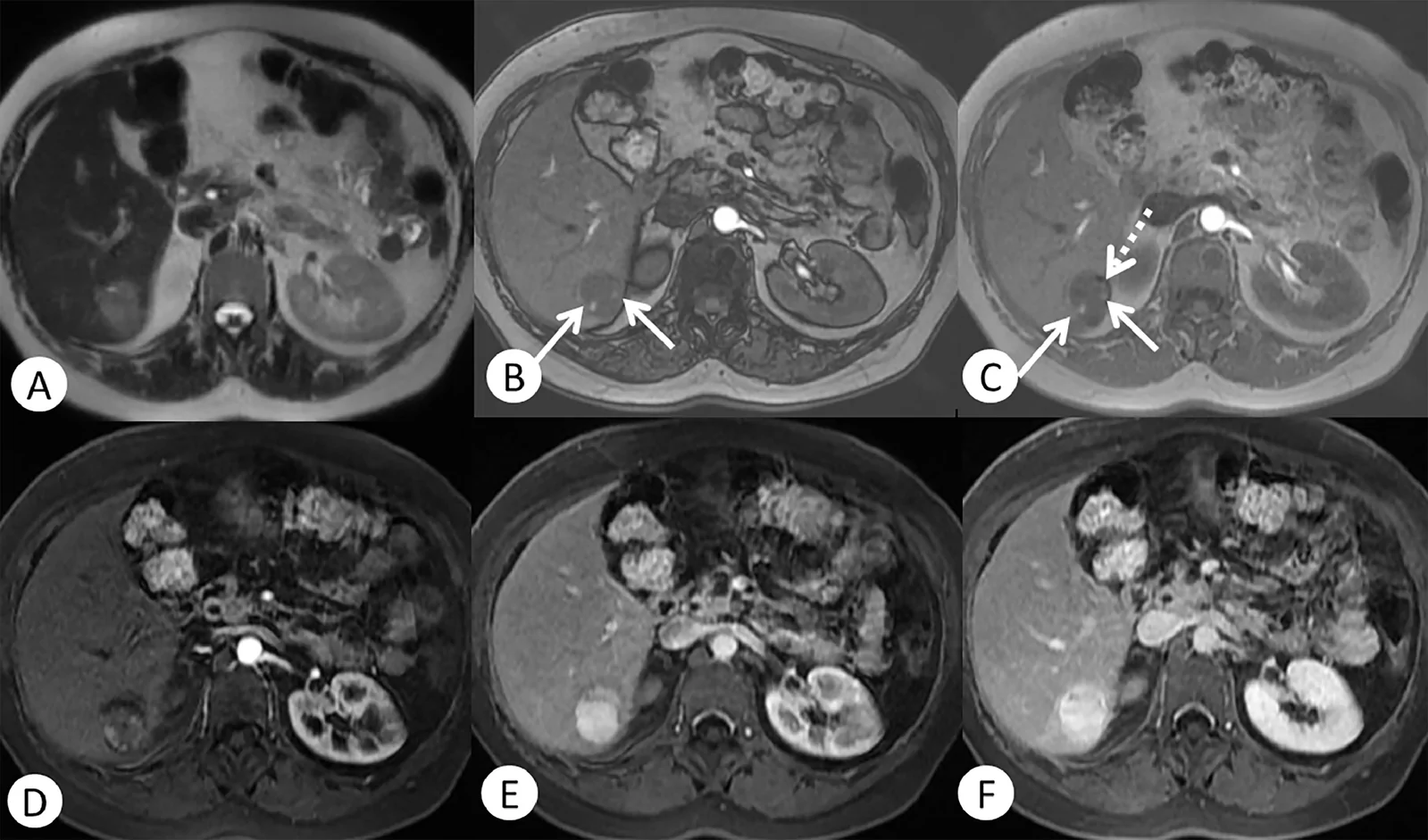
Initial liver MRI evaluation. The lesion demonstrates T2 hyperintensity (A) and low signal intensity on T1-weighted opposed-phase (B) and in-phase gradient-echo (C) images, with small T1-hyperintense foci representing hemorrhagic components (solid arrows). Susceptibility-related signal loss compatible with hemosiderin deposition is seen on the in-phase image (dotted arrow in C). Dynamic contrast-enhanced images show persistent enhancement from the arterial (D) through the portal (E) and delayed phases (F).

**Fig. 5:**
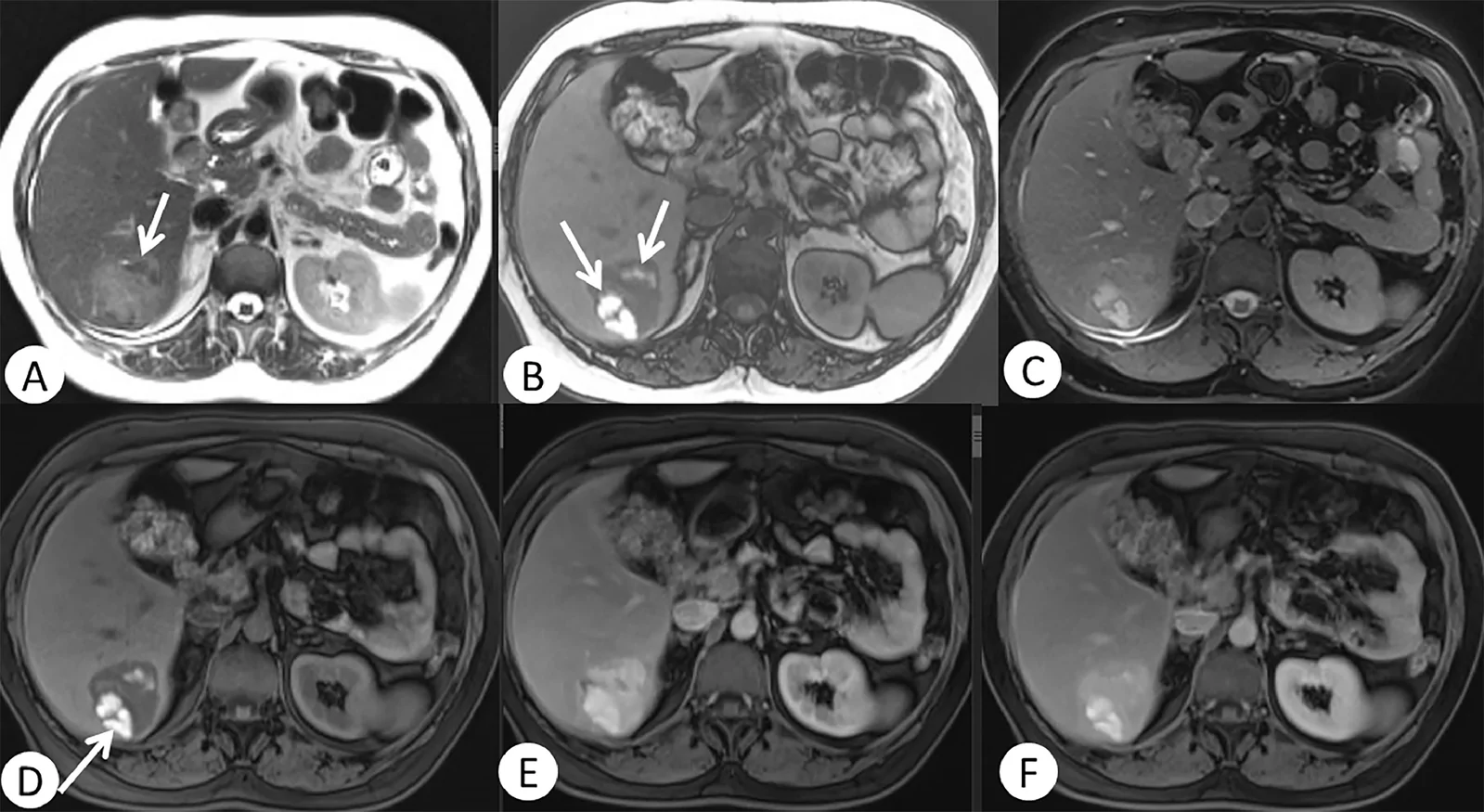
Follow-up liver MRI after 13 months. T2-weighted (A), opposed-phase GRE T1-weighted (B), fat-saturated FIESTA (C), precontrast T1-weighted (D), portal venous (E), and delayed (F) post-gadolinium images demonstrate interval enlargement of the segment VI subcapsular lesion with enlargement and increased conspicuity of internal cystic changes and T1 hyperintense contents compatible with hemorrhagic components (white solid arrows). Persistent enhancement through the portal and delayed phases is again demonstrated (E, F).

Due to the non-specific imaging features, an ultrasound-guided core-needle biopsy was performed. Histopathological evaluation initially suggested a hepatocellular neoplasm, such as hepatocellular adenoma or FNH. However, because the imaging findings were atypical for hepatocellular adenoma, the biopsy specimens were reviewed by an expert hepatobiliary pathologist. The biopsy showed proliferating spindle cells arranged in fascicles with round to oval nuclei, fine chromatin, eosinophilic cytoplasm, and perinuclear vacuoles. Immunohistochemical evaluation showed that the spindle cells were positive for desmin and estrogen receptor (ER), but negative for HMB45, Melan A, DOG1, C-Kit, and SOX10 (Fig. 6). These findings favored a smooth muscle neoplasm of Müllerian origin, and a parasitic leiomyoma was considered. Retrospective review of the CT scan and MRI examinations further supported an extrahepatic/subcapsular origin of the lesion, based on the obtuse angle with the liver capsule. Given the patient's persistent symptoms and diagnostic uncertainty, surgical resection was performed. Histopathologic examination of the resected specimen revealed a subcapsular lesion composed of spindle cells with elongated nuclei and eosinophilic cytoplasm consistent with smooth muscle differentiation. Endometrial glands and stroma were also identified within the lesion, confirming the diagnosis of extrauterine adenomyoma (Fig. 6). The patient had an uneventful postoperative course and remained in good general condition at last follow-up evaluation one year after surgery.

**Fig. 6:**
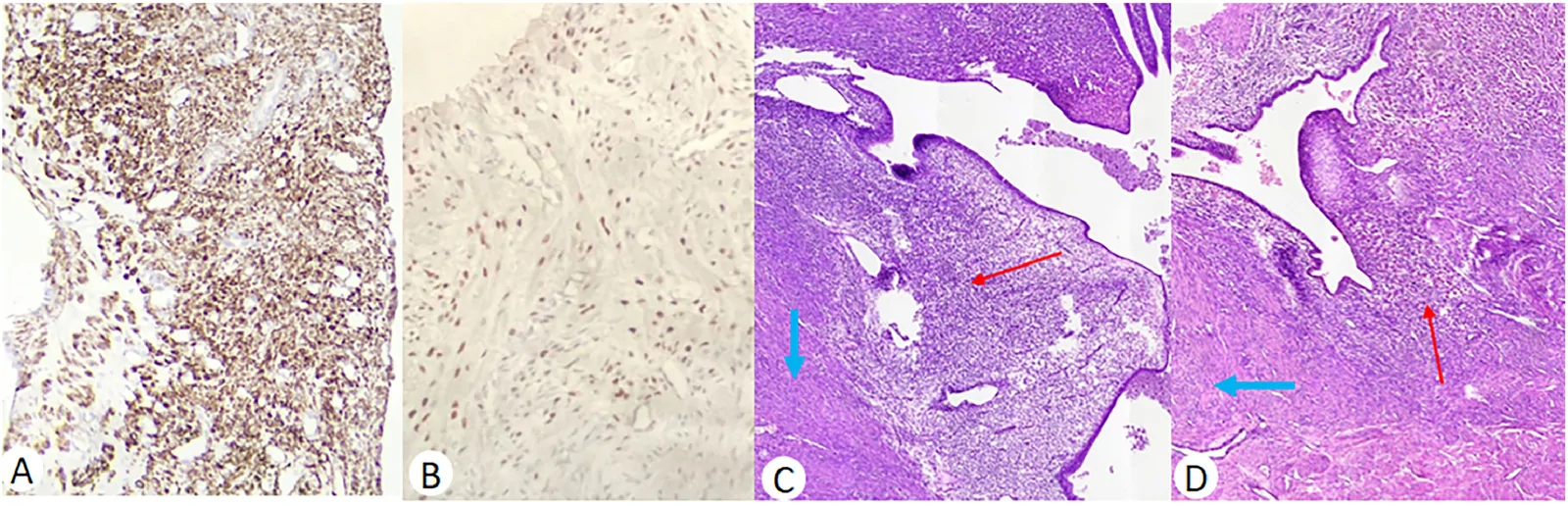
Histopathologic and immunohistochemical findings. (A, B) Desmin and estrogen receptor (ER) positivity, respectively, in the spindle-cell component of the initial core biopsy; HMB45, Melan-A, DOG1, C-Kit, and SOX10 were negative. (C, D) H & E staining of the resected specimen demonstrates smooth muscle bundles (blue arrows) with endometrial glands and stroma (red arrows), confirming extrauterine adenomyoma.

## Discussion

Extrauterine adenomyoma is a rare benign Müllerian lesion composed of benign endometrial glands, endometrial stroma, and smooth muscle bundles. Fewer than 100 cases have been reported in the literature, most occurring in women of reproductive age [2]. The majority arise within the pelvis, including the ovary, pelvic peritoneum, and broad ligament, whereas hepatic or perihepatic involvement is exceedingly rare [[5], [6], [7]]. Because of its rarity and nonspecific clinical and imaging findings, extrauterine adenomyoma is frequently misdiagnosed before surgery.

Clinical manifestations are variable and largely depend on lesion location. Pelvic lesions commonly present with infertility, dyspareunia, dysmenorrhea, and/or persistent pelvic pain, whereas extrapelvic lesions may remain asymptomatic and are often detected incidentally during imaging performed for unrelated indications [8]. In the present case, abdominal pain prompted imaging evaluation; however, the relationship between the patient's symptoms and the hepatic lesion remained uncertain.

Preoperative diagnosis of hepatic extrauterine adenomyoma is particularly challenging because the imaging findings are nonspecific and overlap with those of several benign and malignant hepatic lesions. In the present case, several imaging findings retrospectively suggested an extrahepatic or subcapsular origin rather than a primary hepatic neoplasm. These included the obtuse angle between the lesion and the liver capsule and a small exophytic component extending into Morrison's pouch.

The principal radiologic differential diagnosis includes hepatocellular adenoma, parasitic leiomyoma, endometriotic implants, and other hemorrhagic subcapsular hepatic lesions. Hepatocellular adenomas are typically intraparenchymal hypervascular tumors that demonstrate arterial phase hyperenhancement, frequently followed by variable delayed enhancement depending on the molecular subtype. Although the lesion demonstrated arterial hyperenhancement with persistent delayed enhancement, as well as hemorrhagic components suggestive of hepatocellular adenoma, the subcapsular location and obtuse angle with the liver capsule argued against a primary hepatic neoplasm.

Parasitic leiomyoma represents an important differential diagnosis in women with a history of uterine surgery. It may present as a well-defined extrauterine soft-tissue mass. Unlike extrauterine adenomyoma, however, parasitic leiomyoma is composed exclusively of benign smooth muscle without associated endometrial glands or stroma. Consequently, hemorrhagic and cystic changes are uncommon in parasitic leiomyoma in contrast to extrauterine adenomyoma [9,10]. On MRI, parasitic leiomyomas typically demonstrate homogeneous low T2 signal intensity, whereas extrauterine adenomyomas more commonly exhibit heterogeneous T2 signal intensity and hemorrhagic/cystic changes, as in our case [9,10].

Endometriotic implants may demonstrate intrinsic T1 hyperintensity related to cyclic hemorrhage and occasionally involve the hepatic capsule. However, they are generally considerably smaller than the present lesion and usually lack the abundant smooth muscle component responsible for the persistent enhancement observed in extrauterine adenomyoma. Definitive diagnosis relies on histopathologic examination, demonstrating benign endometrial glands and stroma embedded within smooth muscle bundles. Immunohistochemistry plays an important complementary role. In the present case, the spindle cells were positive for desmin and estrogen receptor and negative for HMB45, Melan A, DOG1, C-Kit, and SOX10, supporting a Müllerian smooth muscle neoplasm while excluding PEComa, gastrointestinal stromal tumor, and other spindle cell neoplasms. The identification of endometrial glands and stroma in the resected specimen ultimately established the diagnosis of extrauterine adenomyoma.

The imaging appearance correlated well with the histopathologic findings. The persistent enhancement likely reflected the abundant smooth muscle component, whereas the progressive T1-hyperintense cystic foci represented recurrent intralesional hemorrhage within ectopic endometrial tissue. In addition, the susceptibility-related signal loss on in-phase GRE images was compatible with hemosiderin deposition secondary to repeated hemorrhagic episodes. These findings provide a plausible explanation for the heterogeneous imaging appearance and the initial diagnostic uncertainty.

Surgical excision remains the treatment of choice because it provides both definitive diagnosis and symptom relief with low recurrence rates. If the lesion is large or associated with endometriosis, more extensive surgery may be required [6]. Hormonal therapies such as progestins, oral contraceptives, and gonadotropin-releasing hormone (GnRH) agonists have been reported in selected patients with unresectable or multifocal disease; however, evidence supporting their efficacy remains limited [11]. Although recurrence is uncommon, it has been reported in some cases, necessitating long-term follow-up.

Hepatic extrauterine adenomyoma is exceedingly rare, and to our knowledge, only four hepatic cases have previously been reported in the literature (Table 1) [[6], [7], [8], [12]]. Despite differences in clinical presentation and gynecologic history, all reported lesions shared several imaging characteristics. The previously reported hepatic cases were predominantly located in the subcapsular aspect of the right hepatic lobe and, when adequately characterized, demonstrated mixed solid-cystic morphology, hemorrhagic components, and persistent enhancement, frequently leading to an initial diagnosis of hepatic adenoma or another hypervascular hepatic lesion. Detailed MRI characterization has been limited in the previously reported hepatic cases, with the most comprehensive imaging description provided by Won et al. [6]. In that case report and our case, MRI demonstrated heterogeneous T2 signal, hemorrhagic changes as high T1 foci, hemosiderin deposition, and persistent enhancement. These findings add to the limited imaging description currently available for hepatic extrauterine adenomyoma. Histopathologic confirmation remains essential for definitive diagnosis [[6], [7], [8], [12]].

**Table 1:** Published cases of hepatic extrauterine adenomyoma and comparison with present case.

Study	Age (years)	Clinical presentation	Gynecologic/clinical context	Location/segment	CT findings	MRI findings	Enhancement pattern	Initial radiologic diagnosis
Wu et al. [12]	29	Low back pain for 2 years, with recent worsening	Laparoscopic myomectomy 5 years earlier	Posterior subcapsular aspect of segment VI of the liver	Hypoechoic mass on US; CT showed a slightly hypodense lesion in the peripheral posterior right hepatic lobe	NR	Heterogeneous enhancement on the arterial phase of CT	Spindle cell tumor; primary or metastatic sarcoma
Tandon et al. [8]	50	Chronic pelvic pain	Pelvic endometriosis	Inferior subcapsular aspect of segment VI	Complex, multiseptated lesion with cystic degeneration and hemorrhagic components	NR	NR	Indeterminate hepatic mass
Luedke et al. [7]	NR	Rapidly enlarging hepatic mass during pregnancy	Pregnancy	Liver; segment NR	NR	NR	NR	Hepatic adenoma
Won et al. [6]	43	Epigastric pain	Previous laparoscopic hysterectomy for uterine leiomyoma	Posterior subcapsular aspect of segment VI	Bilobed subcapsular solid-cystic mass with hemorrhagic foci	Solid component mildly T2 hyperintense; cystic components; hemorrhagic foci	Subtle arterial enhancement with weak, persistent enhancement in the portal and delayed phases, without washout	Hepatic adenoma
Present case	47	Persistent abdominal pain	Previous fibroid surgery; multiple large uterine myomas; two cesarean sections	Posterior subcapsular aspect of segment VI of the liver	Heterogeneous enhancing mass with internal cystic foci; obtuse angle with the liver capsule; small exophytic component	T1 hypointense and T2 hyperintense lesion with hemorrhagic components; hemosiderin deposition; interval enlargement with increasing hemorrhagic/cystic components	Persistent enhancement from arterial through portal and delayed phases; no washout	Hepatocellular adenoma/FNH

NR, not reported.

Our case also illustrates the potential limitations of core needle biopsy in rare Müllerian lesions involving the liver. The initial biopsy favored hepatocellular neoplasms, and expert review subsequently suggested parasitic leiomyoma. Only complete surgical excision with adequate tissue sampling demonstrated the coexistence of smooth muscle, endometrial glands, and endometrial stroma required for a definitive diagnosis. Therefore, radiologic-pathologic correlation is essential whenever imaging findings and biopsy results are discordant.

## Conclusion

Hepatic extrauterine adenomyoma is an exceptionally rare benign Müllerian lesion that may closely mimic hepatocellular adenoma and other hypervascular subcapsular hepatic masses and should be considered in the differential diagnosis of hemorrhagic subcapsular hepatic masses, particularly in women with a history of uterine leiomyomas, adenomyosis, endometriosis, or previous gynecologic surgery. Recognition of characteristic imaging clues—including a subcapsular location, obtuse angle with the liver capsule, an exophytic component—may raise suspicion for an extrahepatic origin, while persistent enhancement and hemorrhagic cystic changes may provide additional clues to the underlying pathology. Nevertheless, histopathologic examination with immunohistochemistry remains essential for definitive diagnosis. Increased awareness of this rare entity and close radiologic-pathologic correlation may reduce diagnostic uncertainty and help to avoid inappropriate management.

## Declaration of generative AI and AI-assisted technologies in the writing process

During the preparation of this work, the author(s) used Gemini to check the grammar and fluency of the text. After using this tool/service, the author(s) reviewed and edited the content as needed and take(s) full responsibility for the content of the published article.

## Ethics approval

This report has been performed in accordance with the Declaration of Helsinki.

## Patient consent

Written informed consents were obtained from the patient for publication of this case report and any accompanying images.
